# Genome-wide assessment of shared genetic landscape of idiopathic pulmonary fibrosis and its comorbidities

**DOI:** 10.1007/s00439-024-02696-9

**Published:** 2024-08-06

**Authors:** Yuanhao Yang, Yong H Sheng, Patricia Carreira, Tong Wang, Huiying Zhao, Ran Wang

**Affiliations:** 1 Mater Research Institute, The University of Queensland, Woolloongabba, QLD Australia; 2 Cancer Program, QIMR Berghofer Medical Research Institute, Herston, QLD Australia; 3 Immunology and Infectious Disease Division, John Curtin School of Medical Research, Australian National University, Acton, ACT Australia; 4 Department of Health Statistics, School of Public Health, Shanxi Medical University, Taiyuan, Shanxi China; 5 Department of Medical Research Center, Sun Yat-sen Memorial Hospital, Sun Yat-sen University, Guangzhou, Guangdong China

## Abstract

Idiopathic pulmonary fibrosis (IPF) is a progressive interstitial lung disease accompanied by both local and systemic comorbidities. Genetic factors play a role in the development of IPF and certain associated comorbidities. Nevertheless, it is uncertain whether there are shared genetic factors underlying IPF and these comorbidities. To bridge this knowledge gap, we conducted a systematic investigation into the shared genetic architecture between IPF and ten prevalent heritable comorbidities (i.e., body mass index [BMI], coronary artery disease [CAD], chronic obstructive pulmonary disease [COPD], gastroesophageal reflux disease, lung cancer, major depressive disorder [MDD], obstructive sleep apnoea, pulmonary hypertension [PH], stroke, and type 2 diabetes), by utilizing large-scale summary data from their respective genome-wide association studies and multi-omics studies. We revealed significant (false discovery rate [FDR] < 0.05) and moderate genetic correlations between IPF and seven comorbidities, excluding lung cancer, MDD and PH. Evidence suggested a partially putative causal effect of IPF on CAD. Notably, we observed FDR-significant genetic enrichments in lung for the cross-trait between IPF and CAD and in liver for the cross-trait between IPF and COPD. Additionally, we identified 65 FDR-significant genes over-represented in 20 biological pathways related to the etiology of IPF, BMI, and COPD, including inflammation-related mucin gene clusters. Several of these genes were associated with clinically relevant drugs for the treatment of IPF, CAD, and/or COPD. Our results underscore the pervasive shared genetic basis between IPF and its common comorbidities and hold future implications for early diagnosis of IPF-related comorbidities, drug repurposing, and the development of novel therapies for IPF.

**Supplementary Information:**

The online version contains supplementary material available at 10.1007/s00439-024-02696-9.

## Introduction

Idiopathic pulmonary fibrosis (IPF) is a rare, chronic, and devastating interstitial lung disease characterised by dry cough and shortness of breath, leading to severe lung scarring and loss of function (Lederer and Martinez 2018). IPF is associated with the histologic and pathologic pattern of usual interstitial pneumonia, including honeycomb lung, dilatation of bronchioles and irregular interlobular septal thickening (Martinez et al. 2017). However, the exact etiology of IPF is unknown and its diagnosis is complicated, requiring collaboration among pulmonologists, radiologists, and pathologists. Due to the complexity of IPF etiology, there are no effective treatments, yielding increasing social and economic burden (Hutchinson et al. 2015). Prior studies revealed frequent co-occurrence of IPF and other respiratory (e.g., chronic obstructive pulmonary disease [COPD], lung cancer, obstructive sleep apnoea [OSA], etc.) and non-respiratory comorbidities (e.g., coronary artery disease gastroesophageal [CAD], gastroesophageal reflux disease [GER], type 2 diabetes [T2D], etc.) (Caminati et al. 2019; Raghu et al. 2015). The impact of comorbidities on the mortality and quality of life of IPF patients is critical (Caminati et al. 2019). For example, the survival rate of IPF patients presenting lung cancer or not taking GER medication (proton pump inhibitor or H2 blocker) is significantly worse (Lee et al. 2011, 2012). These suggest that a better understanding of the relationships between IPF and its comorbidities could improve the quality of life and prolong the survival of IPF patients. Genetic predisposition is associated with the risk of IPF (Allen et al. 2020). Large-scale genome-wide association studies (GWAS) identified thousands of single nucleotide polymorphisms (SNPs) conferring increased susceptibilities to both IPF and its related comorbidities (An et al. 2019; Malik et al. 2018; Rashkin et al. 2020; Strausz et al. 2021; van der Harst and Verweij 2018; Wray et al. 2018; Xue et al. 2018; Yengo et al. 2018), suggesting a potential genetic link between IPF and comorbidities. While previous studies implicated the shared genetics and potential genetic causality between IPF and specific comorbidities (Parcesepe et al. 2023; Zhu et al. 2023), there is still a need for further investigation to uncover the exact genetic basis and relevant biological pathways underlying IPF and its comorbidities.

The advent of GWAS has spurred the development of quantitative genetics approaches (Visscher et al. 2017). Notably, bivariate linkage disequilibrium (LD) score regression (LDSC) and Mendelian randomization (MR) are analytical methods that harness GWAS summary data to estimate genetic correlations and infer potential causal relationships between phenotypes (Bulik-Sullivan et al. 2015; Davies et al. 2018). CPASSOC (cross-phenotype associations) (Zhu et al. 2015) and mBAT-combo (multivariate set-based association test-combo) (Li et al. 2023) are adept at identifying SNPs and candidate genes associated with the susceptibility to a target phenotype or pairs of phenotypes. Additionally, integrating GWAS summary data with other multi-omics information, such as tissue- and cell type-specific gene expression data, as well as functional and biological pathway data, has given rise to additional quantitative genetics approaches like SNPsea (Slowikowski et al. 2014) and ShinyGO (Ge et al. 2020). These tools provide valuable insights into the intricate genetic underpinnings of complex diseases across various multi-omics levels. These advanced approaches have demonstrated high efficacy, yielding successful outcomes that enrich our understanding of the genetic architecture of complex diseases (Xiu et al. 2022; Yang et al. 2023; Zhang et al. 2021). The availability of large-scale GWAS summary data for IPF and its comorbidities, coupled with these advanced analytical tools, enables a comprehensive investigation into the shared genetic architecture between IPF and its comorbidities at multi-omics levels. This, in turn, holds promise for unravelling the genetic foundations of idiopathic diseases, facilitating health management, and enhancing the quality of life for IPF patients.

**Fig. 1:**
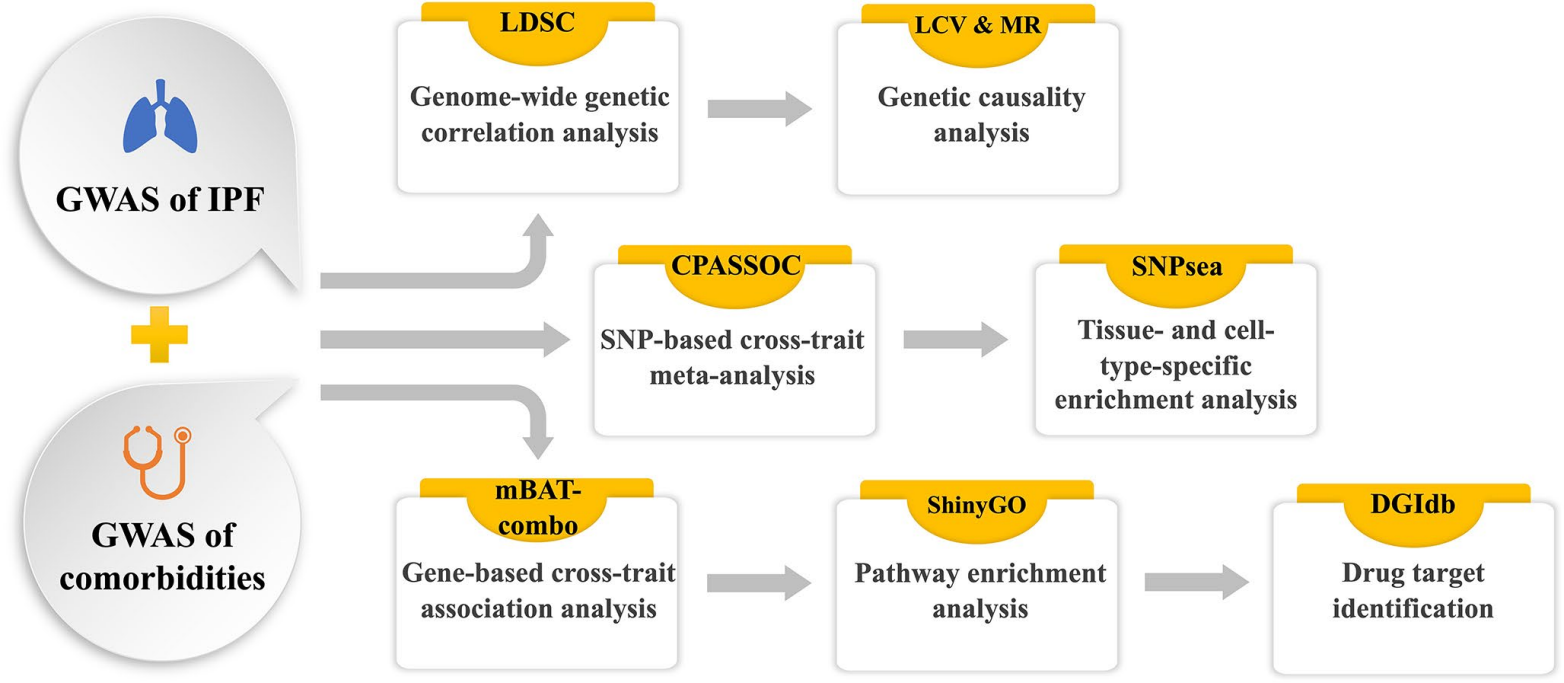
Overview of study design

In this study, we utilized publicly available large-scale GWAS summary data for IPF and ten common comorbidities (Table 1) to evaluate genetic correlations and potential causal relationships between pairs of IPF and these comorbidities (Fig. 1). We identified candidate SNPs and genes likely associated with the susceptibility to both IPF and its comorbidities. Additionally, we unveiled tissue- and cell type-specific enrichments of susceptibility loci associated with the cross-trait between IPF and its comorbidities. Finally, we pinpointed potential drugs and biological pathways implicated in the shared genetics underlying pairs of IPF and its comorbidities.

**Table 1:** GWAS summary data for IPF and ten common comorbidities

Diseases/Traits (Abbreviation)	Publication	Sample size (No. of cases/controls^*^)	No. of SNPs^†^
Idiopathic Pulmonary Fibrosis (IPF)	Wang et al.(Wang et al. 2023)	953,873 (6,257/947,616)	5,786,010
Body Mass Index (BMI)	Yengo et al.(Yengo et al. 2018)	681,275	1,977,062
Coronary Artery Disease (CAD)	Van der Harst et al.(van der Harst and Verweij 2018)	547,261 (122,733/424,528)	6,206,283
Chronic Obstructive Pulmonary Disease (COPD)	Wang et al.(Wang et al. 2023)	995,917 (58,559/937,358)	5,775,999
Gastroesophageal Reflux (GER)	An et al.(An et al. 2019)	385,276 (80,265/305,011)	6,552,681
Lung Cancer	Rashkin et al.(Rashkin et al. 2020)	412,835 (2,485/410,350)	7,430,846
Major Depressive Disorder (MDD)^$^	Wray et al.(Wray et al. 2018)	431,394 (116,404/314,990)	7,247,877
Obstructive Sleep Apnoea (OSA)	Strausz et al.(Strausz et al. 2021)	217,955 (16,761/201,194)	6,902,937
Pulmonary hypertension (PH)	Rhodes et al.(Rhodes et al. 2019)	11,744 (2,085/9,659)	6,876,066
Stroke	Malik et al.(Malik et al. 2018)	446,696 (40,585/406,111)	6,967,429
Type 2 Diabetes (T2D)	Xue et al.(Xue et al. 2018)	659,134 (62,892/596,242)	4,279,624

^*^Number of Cases/Controls are available for diseases/traits of binary phenotypes. ^†^Number of SNPs are filtered out if they were strand-ambiguous, had minor allele frequency < 0.01, had heterogeneity (Cochran’s *Q* test) p-value < 0.05 (specifically for GWAS meta-analysis summary data), or located within the major histocompatibility complex (MHC) region. ^$^GWAS of MDD has excluded samples from UK Biobank. GWAS: genome-wide association study. SNP: single nucleotide polymorphism.

## Materials and methods

### GWAS summary data sources

The IPF associated comorbidities, body mass index (BMI) (Yengo et al. 2018), CAD (van der Harst and Verweij 2018), COPD (Wang et al. 2023), GER (An et al. 2019), lung cancer (Rashkin et al. 2020), major depressive disorder (MDD) (Wray et al. 2018), OSA (Strausz et al. 2021), pulmonary hypertension (PH) (Rhodes et al. 2019), stroke (Malik et al. 2018) and T2D (Xue et al. 2018) were selected based on a systematic literature review (Raghu et al. 2015) and the availability of GWAS summary data. Large-scale GWAS summary data for IPF were also publicly available (Wang et al. 2023). All the GWAS summary data used were generated from European-based cohorts. For each GWAS summary data, SNPs were excluded if they were strand-ambiguous (i.e., A/T, C/G), had minor allele frequency < 0.01, showed heterogeneity p-value < 0.05 (Cochran’s *Q* test for GWAS meta-analysis summary data), or were located within the major histocompatibility complex (MHC) region (chromosome 6: 28477797–33448354). Consequently, approximately 2–7 million common SNPs per GWAS were selected for downstream analyses. Details of each GWAS summary data are summarised in Table 1.

### Single-trait heritability and cross-trait genetic correlation analysis

Single-trait liability-scale heritability (*h*^*2*^) and cross-trait genetic correlation (*r*_*g*_) between IPF and each comorbidity were conducted using univariate and bivariate LDSC (Bulik-Sullivan et al. 2015), respectively, without intercept constrained. The LDSC model measures the trait heritability and genetic correlation between traits by quantifying the slope of the model that regresses GWAS test statistics (e.g., *Z*-score) against the LD score per SNP. Pre-computed LD scores of the 1000 Genomes European reference panel were applied for each LDSC analysis. For genetic correlation, significance was determined if surpassing the Benjamini–Hochberg false discovery rate (FDR) < 0.05.

### Genetic causality analysis

Latent causal variable (LCV) (O’Connor and Price 2018) was applied to assess the putative causal relationship between traits. IPF-comorbidity pairs with an FDR significant (FDR < 0.05) genetic correlation were selected for LCV analysis. LCV model assumes that a significant genetic correlation between traits can be mediated by a latent ‘causal’ variable, which capably distinguishes partial causality from pleiotropy (Davies et al. 2018). LCV model estimates the magnitude of the latent ‘causal’ mediator using the mean posterior genetic causal proportion (GCP), which is a numerical value ranging from 0 (no causal relationship) to 1 (fully causal relationship), with a higher value indicating evidence for a more robust partially genetic causality. In our study, LCV analysis was performed according to the same 1000 Genomes European reference panel used in the LDSC analysis. Significant (partial) genetic causality was declared as those surpassing the FDR < 0.05. A strong genetic causal relationship was further determined with an estimated GCP greater than 0.60.

### Mendelian randomisation analysis

Multiple bi-directional MR approaches (Davies et al. 2018) were applied to the pairs of traits with a (partial) genetic causal relationship suggested by LCV to examine the reliability of the significant (partial) genetic causality and measure the magnitude of the (partial) causal effect between IPF and specific comorbidity. MR is a statistical approach using SNPs as instrumental variables for causal inference, which is equivalent to a random allocation in randomised controlled trials (Davies et al. 2018). Six two-sample MR models with their specific assumptions on pleiotropy were used to evaluate the potential causal relationships between IPF and comorbidity, including IVW (inverse variance weighting) (Burgess et al. 2013), MR-Egger (Burgess and Thompson 2017), weighted median (Bowden et al. 2016), weighted mode (Hartwig et al. 2017), GSMR (generalised summary-data-based Mendelian randomisation) (Zhu et al. 2018) and CAUSE (Causal analysis using summary effect estimates) (Morrison et al. 2020). IPF-comorbidity relationships were thought to be trustworthy and less likely to be influenced by pleiotropy if they were identified consistently and significantly across all MR models. MR models were carried out through the R packages ‘*TwoSampleMR*’, ‘*gsmr*’ and ‘*cause*’ with instrumental variables selected from independent (LD *r*^*2*^ < 0.05 within 1,000-kb windows) exposure-associated SNPs with p-value < 5 × 10^− 8^ (with exception to CAUSE that applied p-value < 1 × 10^− 3^). The estimated MR effects were converted from logit-scale to liability-scale and then transformed to odds ratios (ORs) (Byrne et al. 2020).

### SNP-based cross-trait meta-analysis

After exploring genetic correlations and putative causal relationships between IPF and its comorbidities, CPASSOC (Zhu et al. 2015) was implemented to detect candidate SNPs associated with risks for IPF and specific comorbidity (cross-trait). CPASSOC estimates the cross-trait associations by combining the GWAS summary data across two traits through a sample size-weighted meta-analysis, which could allow for the potential genetic heterogeneity effects and sample overlap between GWAS (Yang et al. 2021; Zhu et al. 2015). CPASSOC was conducted for specific pairs of IPF and its comorbidities with significant genetic correlation. The previously unreported cross-trait associated SNPs were defined if (1) they were independently (after LD clumping with LD *r*^*2*^ < 0.05 within 1,000-kb windows) and genome-wide significantly associated (*p* < 5 × 10^− 8^) with cross-trait (e.g., IPF-BMI) but not the original single-trait (e.g., IPF, BMI), and (2) they had a low LD (*r*^*2*^ < 0.05) with genome-wide significant loci previously identified in the original single-trait GWAS.

### Gene atlas tissue and cell-type specific enrichment analysis

For specific IPF-comorbidity pairs with an FDR significant (FDR < 0.05) genetic correlation, enrichment analysis was applied through SNPsea (Slowikowski et al. 2014) to assess whether there was significant enrichment in specific tissues and cell types for the susceptibility loci of the cross-trait CPASSOC summary data of these trait pairs (e.g., IPF-BMI). The human gene atlas from the Genomics Institute of the Novartis Research Foundation (Su et al. 2004) was utilised as the gene expression reference, which encompasses expression profiles of 79 human tissues and cell types. Among them, 58 tissues and cell types were selected for the downstream analyses, after excluding those based on embryo, related to the reproductive system, and related to leukemia, primarily due to their distinct patterns of gene expression and pathway activations, which differ from those underpinning normal biological processes in somatic tissues and cell types (Assou et al. 2011; Chiorazzi and Ferrarini 2011). Genome-wide significant (*p* < 5 × 10^− 8^) and suggestive significant SNPs (*p* < 1 × 10^− 5^) of the CPASSOC summary data were selected as the input of the susceptibility loci, designating for the primary analysis and sensitivity analysis, respectively. For a given SNP set (e.g., genome-wide significant loci of the cross-trait IPF-BMI), SNPsea evaluates whether it (in aggregation) shows enrichment in specific tissues and cell types, by comparing its aggregated score with the aggregated score generated by a random SNP set after matching the SNP set size. To obtain more accurate results, the maximum number of iterations for comparing the target SNP set and randomly matched SNP set were set at 1 million. Significant enrichments were identified as those surpassing the FDR < 5% after correcting for multiple comparisons.

### Gene-based cross-trait association analysis

Next, the analyses were extended from the SNP level to the gene level by applying mBAT-combo (Li et al. 2023) to identify candidate genes involved in the shared genetics between pairs of IPF and its comorbidities with an FDR significant genetic correlation. In brief, mBAT-combo maps SNPs from GWAS summary data to a gene unit if SNPs were located within 50 kb upstream or downstream of the gene length boundary; mBAT-combo then evaluates the strengths of associations between genes and target traits (e.g., IPF, CAD) by combining mBAT and fastBAT statistics using aggregated Cauchy combination. The mBAT statistics are calculated through a non-zero variance estimation approach that can correct the SNPs with masking effects (i.e., SNPs in high positive LD have a negative genetic covariance, and vice versa), whereas the fastBAT statistics are based on the sum $\:{\chi\:}^{2}$ method. In our study, mBAT-combo was performed for 19,528 genes (after excluding genes located within the MHC region) with LD structure adjusted. The candidate pleiotropic genes associated with pairs of IPF, and its comorbidities were determined as those surpassing the FDR 5% p-value threshold for both traits.

### Identification of drug-gene interactions

The shared risk genes associated with pairs of IPF and its specific comorbidities, as identified by mBAT-combo, were then examined through the Drug-Gene Interaction database (DGIdb; https://www.dgidb.org/) (Freshour et al. 2021), to explore their potential associations with known clinically relevant drugs. The DGIdb is a comprehensive and publicly available resource that extrapolates insights into drug-gene interactions by synthesizing data from scientific literature, clinical trials, and pharmacological databases. Significant drug-gene interactions were determined if they were involved in well-established pathways and molecular functions and have been reported in at least one prior research study, which may have the potential for identifying novel drugs for the treatment of IPF and its specific comorbidities.

### Pathway enrichment analysis

To discover potential biological pathways involved in the increased risk for the co-occurred IPF and comorbidities, pathway enrichment analysis was performed via ShinyGO (Ge et al. 2020) according to the mBAT-combo-based candidate gene sets identified for each pair of IPF and its comorbidity. ShinyGO was applied based on Gene Ontology (GO) annotation resource, including biological processes, cellular components, and molecular function. Significantly enriched pathways for each pair of traits were selected as those reporting with ≥ 2 gene annotations and surpassing the 5% FDR significance level.

## Results

### Pervasive genetic correlations between IPF and comorbidities

**Fig. 2:**
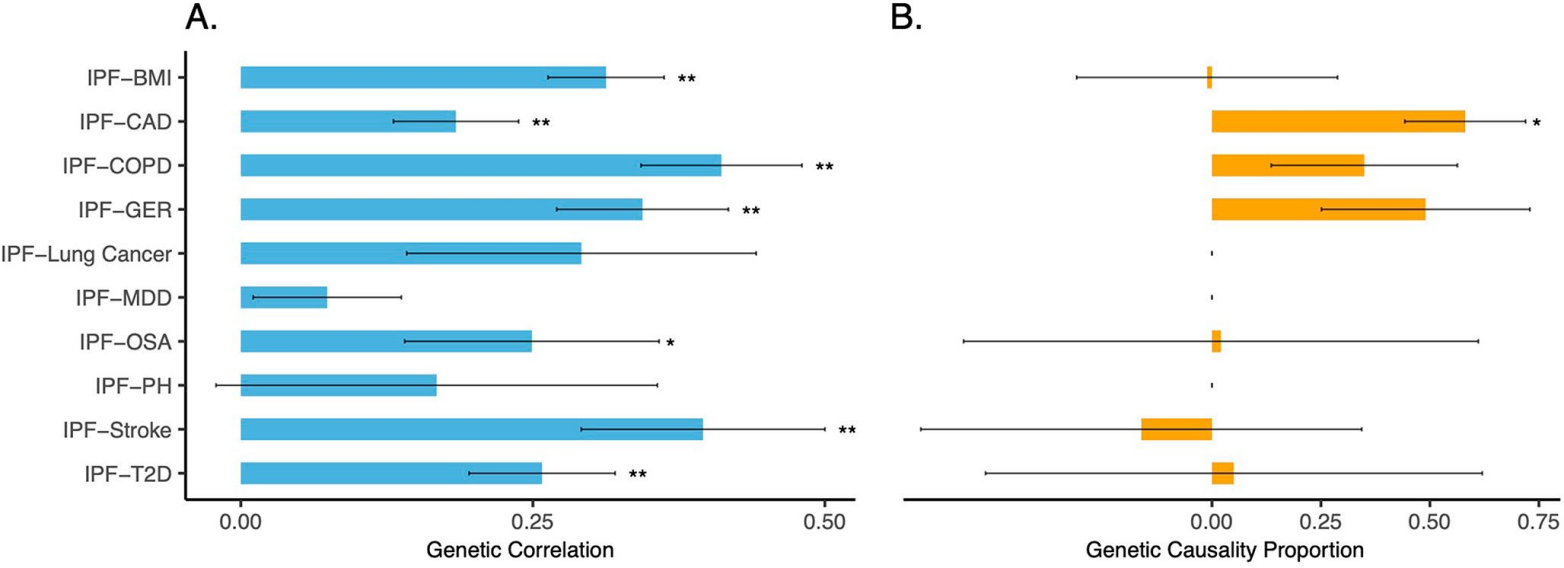
Estimated (**A**) genetic correlations (*r*_*g*_, left, colour in blue) and (**B**) genetic causality proportions (GCP, right, colour in orange) between IPF and comorbidities using LDSC and LCV, respectively. LCV results are only available for trait pairs with FDR significant *r*_*g*_. Positive GCP suggests a partial causality of IPF on specific comorbidity, and vice versa. Error bars represent the standard error of the estimated *r*_*g*_/GCP. The single asterisk (^*^) and double asterisk (^**^) indicate if the estimated *r*_*g*_/GCP surpassed the FDR 5% and Bonferroni-corrected significance level, respectively. LDSC: linkage disequilibrium score regression. LCV: latent causal variable model. IPF: idiopathic pulmonary fibrosis. BMI: body mass index. CAD: coronary artery disease. COPD: chronic obstructive pulmonary disease. GER: gastroesophageal reflux. MDD: major depressive disorder. OSA: obstructive sleep apnoea. PH: pulmonary hypertension. T2D: type 2 diabetes. FDR: Benjamini–Hochberg false discovery rate

The SNP-based liability-scale heritability for each individual trait and the genetic correlations between IPF and its comorbidities were estimated using LDSC. Our findings revealed significant liability-scale heritability for all traits (Table S1) and FDR significant genetic correlations between IPF and seven (out of the ten) comorbidities (Fig. 2A and Table S1). Six of these comorbidities exhibited stringent Bonferroni-corrected significance levels (*p* < 5 × 10^− 3^ = 0.05/10) with IPF, including BMI (*r*_*g*_ = 0.31, *p* = 3.09 × 10^− 10^), CAD (*r*_*g*_ = 0.18, *p* = 6.00 × 10^− 4^), COPD (*r*_*g*_ = 0.41, *p* = 2.33 × 10^− 9^), GER (*r*_*g*_ = 0.34, *p* = 2.90 × 10^− 6^), stroke (*r*_*g*_ = 0.40, *p* = 1.00 × 10^− 4^), and T2D (*r*_*g*_ = 0.26, *p* = 3.71 × 10^− 5^), except for OSA (*r*_*g*_ = 0.25, *p* = 0.02). These results suggest substantial contributions from shared genetic factors to the development of IPF and the majority of the comorbidities investigated. This emphasizes the interconnected genetic underpinnings between IPF and various associated health conditions.

### Suggestive evidence for a partial genetic causality of IPF on CAD

Leveraging the LCV methodology, we explored whether the observed genetic correlations, particularly those found to be FDR significant, could be attributed to causality and/or pleiotropy. The LCV analyses unveiled a partial genetic causality of IPF on CAD (GCP = 0.58, *p* = 1.10 × 10^− 15^; Fig. 2B). However, in contrast, the LCV model showed no compelling evidence of genetic causality for the remaining six IPF-comorbidity pairs (Fig. 2B and Table S2). These outcomes strongly suggest that, in the majority of cases, the observed genetic correlations between IPF and its comorbidities are predominantly driven by pleiotropy rather than causality.

Given the partial genetic causality of IPF influencing CAD as indicated by LCV, we proceeded to estimate the magnitude of this partial causal effect using six bi-directional two-sample MR models. Three MR models (IVW, weighted median, and GSMR) demonstrated nominally significant (*p* < 0.05) causal effect of IPF on CAD, with the estimated liability-scale odds ratios (ORs) ranging from 1.08 to 1.38 (Table 2). Conversely, the remaining MR models (MR-Egger, weighted mode, and CAUSE) did not yield nominally significant results. Taken together, the collective evidence from multiple MR models and the LCV model suggests that the genetic overlap between IPF and CAD is likely explained by a combination of both pleiotropy and partial causality.

**Table 2:** Causal relationship (in ORs) between IPF and CAD estimated from multiple MR models

MR model	IPF as exposure, CAD as outcome				CAD as exposure, IPF as outcome			
	No. SNPs	OR (observed level)	OR (liability level)^*^	*p*-value	No. SNPs	OR (observed level)	OR (liability level)^*^	*p*-value
IVW	10	1.03	1.08	0.03	197	0.97	0.99	0.33
MR-Egger	10	1.04	1.09	0.46	197	1.02	1.01	0.73
Weighted median	10	1.04	1.09	0.05	197	0.96	0.98	0.42
Weighted mode	10	1.05	1.13	0.15	197	0.94	0.98	0.37
GSMR	10	1.14	1.38	0.03	195	0.99	1.00	0.28
CAUSE	993	1.01	1.01	0.70	2952	1.01	1.01	0.64

^*^ORs in liability scale are calculated using Byrne et al.(Byrne et al. 2020) method, assuming the population prevalence for IPF and CAD were 0.05%(Fernandez Perez et al. 2010) and 0.12%(Ralapanawa and Sivakanesan 2021), respectively. MR: Mendelian randomisation. IVW: inverse variance weighting. GSMR: generalised summary-data-based Mendelian randomisation. CAUSE: causal analysis using summary effect estimates. OR: odds ratio. IPF: idiopathic pulmonary fibrosis. CAD: coronary artery disease.

### Cross-trait SNPs associated with the shared genetics between IPF and specific comorbidities

Subsequently, employing CPASSOC, we aimed to pinpoint risk SNPs associated with the cross-trait pairs exhibiting FDR significant genetic correlations, as determined by LDSC. This analysis revealed a total of 26 independent and previously unreported genome-wide significant loci (Table 3). Notably, these SNPs were identified as shared risk factors between IPF and five specific comorbidities, encompassing BMI (*n* = 18), CAD (*n* = 2), COPD (*n* = 1), GER (*n* = 3), and T2D (*n* = 2). Among these loci, two SNPs, namely rs10947563 (IPF-BMI specific; chromosome 6: 35653437) and rs62402061 (IPF-GER specific; chromosome 6: 35381192), exhibited a mild linkage disequilibrium (*r*^*2*^ = 0.12), suggesting the existence of a shared genetic region influencing both IPF and the comorbidities BMI and GER. Beyond these 26 previously unreported SNPs, our analysis identified a substantial number of SNPs showing genome-wide significant associations with cross-trait pairs (Table S3). The majority of these SNPs (> 90%) were primarily driven by the genome-wide significant loci associated explicitly with the original single traits, underscoring the importance of these specific genetic regions in the observed cross-trait associations.

**Table 3:** Previously unreported SNPs associated with cross-trait IPF and specific comorbidities identified by CPASSOC

Trait pair	SNP	CHR	BP	EA	RA	EAF	*p*-value (CPASSOC)	*p*-value (IPF)	*p*-value (comorbidity)
IPF-BMI	rs11576725	1	208,706,936	A	G	0.30	4.56 × 10^− 8^	0.34	6.20 × 10^− 8^
IPF-BMI	rs6550052	3	31,446,413	C	A	0.31	3.70 × 10^− 8^	5.00 × 10^− 4^	3.40 × 10^− 6^
IPF-BMI	rs1403846	4	119,101,723	C	T	0.19	4.63 × 10^− 8^	0.46	6.40 × 10^− 8^
IPF-BMI	rs869090	4	131,023,284	A	G	0.41	3.10 × 10^− 8^	0.29	5.10 × 10^− 8^
IPF-BMI	rs1225598	6	28,160,799	G	T	0.23	1.43 × 10^− 8^	4.46 × 10^− 5^	4.90 × 10^− 5^
IPF-BMI	rs10947563	6	35,653,437	A	G	0.72	4.28 × 10^− 10^	3.56 × 10^− 5^	7.10 × 10^− 7^
IPF-BMI	rs7796645	7	24,468,285	G	A	0.38	3.27 × 10^− 8^	2.07 × 10^− 3^	1.90 × 10^− 7^
IPF-BMI	rs11558476	7	99,654,689	A	G	0.27	5.99 × 10^− 9^	2.05 × 10^− 7^	6.00 × 10^− 3^
IPF-BMI	rs2979655	8	6,329,470	G	T	0.16	4.69 × 10^− 8^	0.37	6.30 × 10^− 8^
IPF-BMI	rs2272724	8	124,142,489	C	T	0.36	1.68 × 10^− 10^	7.29 × 10^− 5^	7.00 × 10^− 8^
IPF-BMI	rs10808546	8	126,495,818	T	C	0.45	3.64 × 10^− 8^	0.36	5.20 × 10^− 8^
IPF-BMI	rs7043482	9	85,135,915	C	A	0.34	4.47 × 10^− 8^	0.92	6.70 × 10^− 8^
IPF-BMI	rs4944000	11	87,459,879	A	G	0.28	4.56 × 10^− 8^	0.51	6.70 × 10^− 8^
IPF-BMI	rs2657300	12	116,957,424	G	A	0.52	4.47 × 10^− 8^	0.06	7.40 × 10^− 8^
IPF-BMI	rs1278769	13	113,536,627	G	A	0.76	4.07 × 10^− 8^	2.96 × 10^− 5^	3.30 × 10^− 4^
IPF-BMI	rs225882	14	30,480,123	T	C	0.72	5.97 × 10^− 9^	6.91 × 10^− 4^	6.30 × 10^− 8^
IPF-BMI	rs12933086	16	9,225,494	G	A	0.28	3.29 × 10^− 8^	0.45	5.30 × 10^− 8^
IPF-BMI	rs6095604	20	48,287,029	T	C	0.53	3.88 × 10^− 8^	4.48 × 10^− 4^	6.40 × 10^− 6^
IPF-CAD	rs1498399	1	77,947,239	G	A	0.39	1.52 × 10^− 8^	1.91 × 10^− 5^	2.20 × 10^− 5^
IPF-CAD	rs9803935	1	150,552,622	G	T	0.56	3.14 × 10^− 8^	3.60 × 10^− 4^	1.76 × 10^− 7^
IPF-COPD	rs10461165	4	89,770,583	A	G	0.25	5.75 × 10^− 9^	2.55 × 10^− 4^	9.97 × 10^− 6^
IPF-GER	rs3757132	6	25,832,691	T	C	0.26	1.05 × 10^− 8^	1.32 × 10^− 7^	6.48 × 10^− 3^
IPF-GER	rs9689160	6	34,749,849	G	A	0.27	1.44 × 10^− 8^	1.25 × 10^− 5^	1.84 × 10^− 6^
IPF-GER	rs62402061	6	35,381,192	A	G	0.08	2.07 × 10^− 8^	3.22 × 10^− 6^	5.81 × 10^− 5^
IPF-T2D	rs9853081	3	71,531,170	G	A	0.48	5.78 × 10^− 9^	2.94 × 10^− 5^	4.71 × 10^− 6^
IPF-T2D	rs7164289	15	40,880,865	A	G	0.15	1.88 × 10^− 9^	5.70 × 10^− 6^	3.23 × 10^− 5^

CPASSOC: Cross Phenotype Association. SNP: single nucleotide polymorphism. CHR: chromosome. BP: base pair position. EA: effect allele. RA: reference allele. EAF: effect allele frequency. IPF: idiopathic pulmonary fibrosis. BMI: body mass index. CAD: coronary artery disease. COPD: chronic obstructive pulmonary disease. GER: gastroesophageal reflux. T2D: type 2 diabetes.

### Tissue-specific enrichment for susceptibility loci of cross-trait between IPF and specific comorbidity

**Fig. 3:**
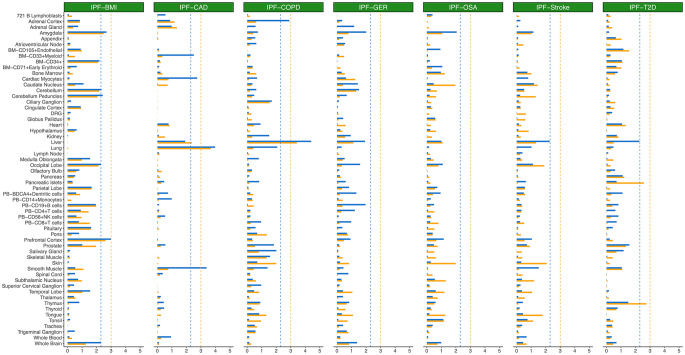
Gene atlas tissue- and cell-type-specific enrichment for the seven cross-trait pairs (i.e., IPF-BMI, IPF-CAD, IPF-COPD, IPF-GER, IPF-OSA, IPF-Stroke and IPF-T2D) according to their CPASSOC summary data. Negative log10 p-values are displayed on the x-axis. The orange and dodger blue bars represent the results using genome-wide significant SNPs with p-values < 5 × 10^− 8^ and suggestive significant SNPs with p-values < 1 × 10^− 5^, with the same-coloured and dotted lines for the FDR threshold < 5% for multiple comparisons. IPF: idiopathic pulmonary fibrosis. BMI: body mass index. CAD: coronary artery disease. COPD: chronic obstructive pulmonary disease. GER: gastroesophageal reflux. OSA: obstructive sleep apnoea. T2D: type 2 diabetes. FDR: Benjamini–Hochberg false discovery rate

Utilizing the cross-trait summary data derived from CPASSOC, we applied SNPsea to explore whether susceptibility loci for each cross-trait were enriched in specific tissues and cell types. Our analysis revealed FDR significant enrichments in the lung for the cross-trait IPF-CAD and in the liver for the cross-trait IPF-COPD (Fig. 3and Table S4). Notably, these enrichments remained significant regardless of whether genome-wide significant SNPs (*p* < 5 × 10^− 8^) or suggestive significant SNPs (*p* < 1 × 10^− 5^) were utilized as the input for susceptibility loci. Expanding beyond these prominent enrichments, when considering only the suggestive significant SNPs as the input, we observed FDR significant enrichments for the cross-trait IPF-CAD in tissues and cell types associated with the cardiovascular system, including cardiac myocytes, smooth muscle, and CD33^+^ cells in myeloid. Similarly, for the cross-trait IPF-COPD, significant enrichment was identified in the adrenal cortex. Furthermore, for the cross-trait IPF-BMI, significant enrichments were found in various brain regions, such as the amygdala, cerebellum, cerebellum peduncles, occipital lobe, prefrontal cortex, and the whole brain. Conversely, the enrichment results for the cross-trait pairs IPF-GER, IPF-OSA, IPF-Stroke, and IPF-T2D were universally non-significant across all tissues or cell types examined. This suggests a nuanced tissue-specificity in the genetic associations between IPF and its comorbidities, underscoring the importance of considering tissue context in understanding the shared genetic architecture of these conditions.

### Cross-trait genes associated with the shared genetics between IPF and specific comorbidities

**Fig. 4:**
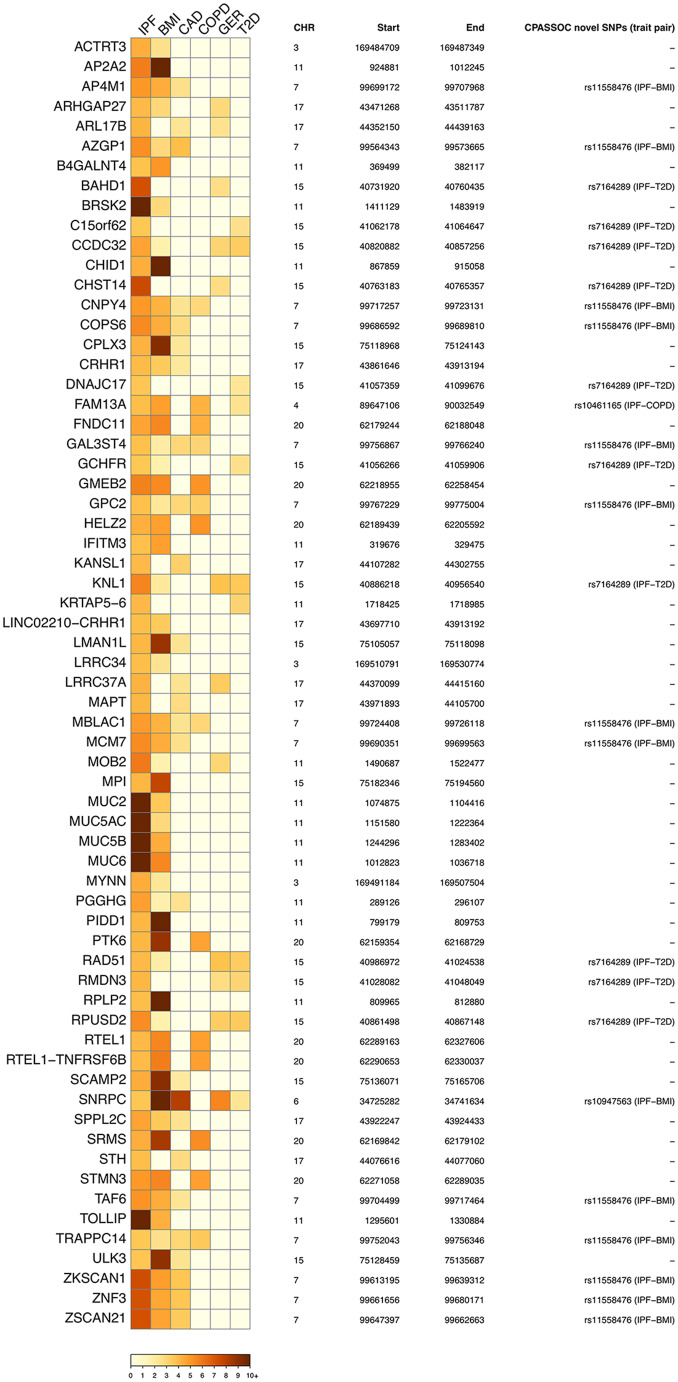
Significant candidate genes shared between specific pairs of IPF and comorbidity. Significant genes are determined by surpassing the 5% FDR significance level for both IPF and at least one comorbidity and displayed in the heatmap (left) by the negative log10 p-value according to the mBAT-combo models. Candidate gene location information and their interactions with ‘novel’ cross-trait SNPs (within 1,000-kb windows) identified by CPASSOC analysis are displayed on the right. FDR: Benjamini–Hochberg false discovery rate. IPF: idiopathic pulmonary fibrosis. BMI: body mass index. CAD: coronary artery disease. COPD: chronic obstructive pulmonary disease. GER: gastroesophageal reflux. T2D: type 2 diabetes. CHR: chromosome. CPASSOC: Cross Phenotype Association

Subsequently, transitioning from the SNP level to the gene level, we employed mBAT-combo to identify candidate genes shared between IPF and its comorbidities exhibiting FDR significant genetic correlations. Our analysis revealed 65 candidate genes with FDR significant associations between IPF and specific comorbidities (Fig. 4 and Table S5), including BMI (*n* = 54), CAD (*n* = 26), COPD (*n* = 14), GER (*n* = 12), and T2D (*n* = 11). Of these candidate genes, 40 were found to be implicated in the risks of IPF and two or more comorbidities. Remarkably, ten genes - *CCDC32*,* CNPY4*,* FAM13A*,* GAL3ST4*,* GPC2*,* KNL1*,* MBLAC1*,* RAD51*,* RPUSD2*, and *TRAPPC14 -* exhibited significant genetic associations with IPF and three comorbidities. The gene *SNRPC* was identified as a regulator of risks for IPF and four comorbidities (BMI, CAD, GER, and T2D). Moreover, our analysis identified 25 genes that either contained or were located close to at least one previously unreported cross-trait associated SNP (identified by the CPASSOC analysis; Tables 3 and Fig. 4). The majority of these genes (24/25) were specific to the IPF-BMI cross-trait (on chromosomes 6 and 7) or the IPF-T2D cross-trait (on chromosome 15). The remaining gene, *FAM13A*, was specific to the IPF-COPD cross-trait on chromosome 4. These findings highlight a subset of genes potentially playing a pivotal role in the shared genetic architecture between IPF and its comorbidities.

### Potential drug-gene interactions associated with pairs of IPF and specific comorbidities

Among the 65 FDR significant genes associated with pairs of IPF and specific comorbidities, as identified by mBAT-combo, six genes (*BRSK2*,* CRHR1*,* MAPT*,* PTK6*,* ULK3*,* and MUC2*) were further found to be associated with potential drugs (Table 4). Four of these genes (*CRHR1*,* MAPT*,* ULK3*, and *MUC2*) were found to interact with multiple drugs (≥ 2), while the remaining two genes (*BRSK2* and *PTK6*) each interacted with one drug. In detail, a total of 14 drugs were associated with these six genes. Five drugs, interacted by *BRSK2*, *CRHR1*, and *ULK3*, were overlapped between IPF-BMI and IPF-CAD. One drug, interacted by *PTK6*, was overlapped between IPF-BMI and IPF-COPD. Three drugs, interacted by *MUC2*, were specific to IPF-BMI; and five drugs, interacted by *MAPT*, were specific to IPF-CAD. Importantly, no previously reported drug-gene interactions were observed for either IPF-GER or IPF-T2D. These findings highlight potential therapeutic avenues and drug repurposing opportunities by targeting genes associated with both IPF and its comorbidities, particularly for obesity and coronary heart disease.

**Table 4:** Potential drugs associated with the shared candidate genes underlying IPF and specific comorbidities

Trait pair	Gene	Drug name	Sources^*^
IPF-BMI	*BRSK2*	HESPERADIN	DTC
IPF-BMI	*CRHR1*	BUDESONIDE	PharmGKB
IPF-BMI	*CRHR1*	TRIAMCINOLONE	PharmGKB
IPF-BMI	*CRHR1*	FLUOXETINE	PharmGKB
IPF-BMI	*PTK6*	SB-202,190	DTC
IPF-BMI	*ULK3*	HESPERADIN	DTC
IPF-BMI	*ULK3*	IMATINIB	PharmGKB
IPF-BMI	*MUC2*	FLUOROURACIL	NCI
IPF-BMI	*MUC2*	CHEMBL35482	NCI
IPF-BMI	*MUC2*	DECITABINE	NCI
IPF-CAD	*CRHR1*	BUDESONIDE	PharmGKB
IPF-CAD	*CRHR1*	TRIAMCINOLONE	PharmGKB
IPF-CAD	*CRHR1*	FLUOXETINE	PharmGKB
IPF-CAD	*MAPT*	CHEMBL2181040	DTC
IPF-CAD	*MAPT*	CHEMBL2181046	DTC
IPF-CAD	*MAPT*	CHEMBL2181039	DTC
IPF-CAD	*MAPT*	CHEMBL2181041	DTC
IPF-CAD	*MAPT*	LANSOPRAZOLE	DTC
IPF-CAD	*ULK3*	HESPERADIN	DTC
IPF-CAD	*ULK3*	IMATINIB	PharmGKB
IPF-COPD	*PTK6*	SB-202,190	DTC

^*^Sources are based on the drug-gene interaction database (DGIdb; https://www.dgidb.org/) and supported by previous research studies. IPF: idiopathic pulmonary fibrosis. BMI: body mass index. CAD: coronary artery disease. COPD: chronic obstructive pulmonary disease. DTC: Drug Target Commons. PharmGKB: The Pharmacogenomics Knowledgebase. NCI: National Cancer Institute.

### Shared biological pathways underline IPF and specific comorbidities

**Fig. 5:**
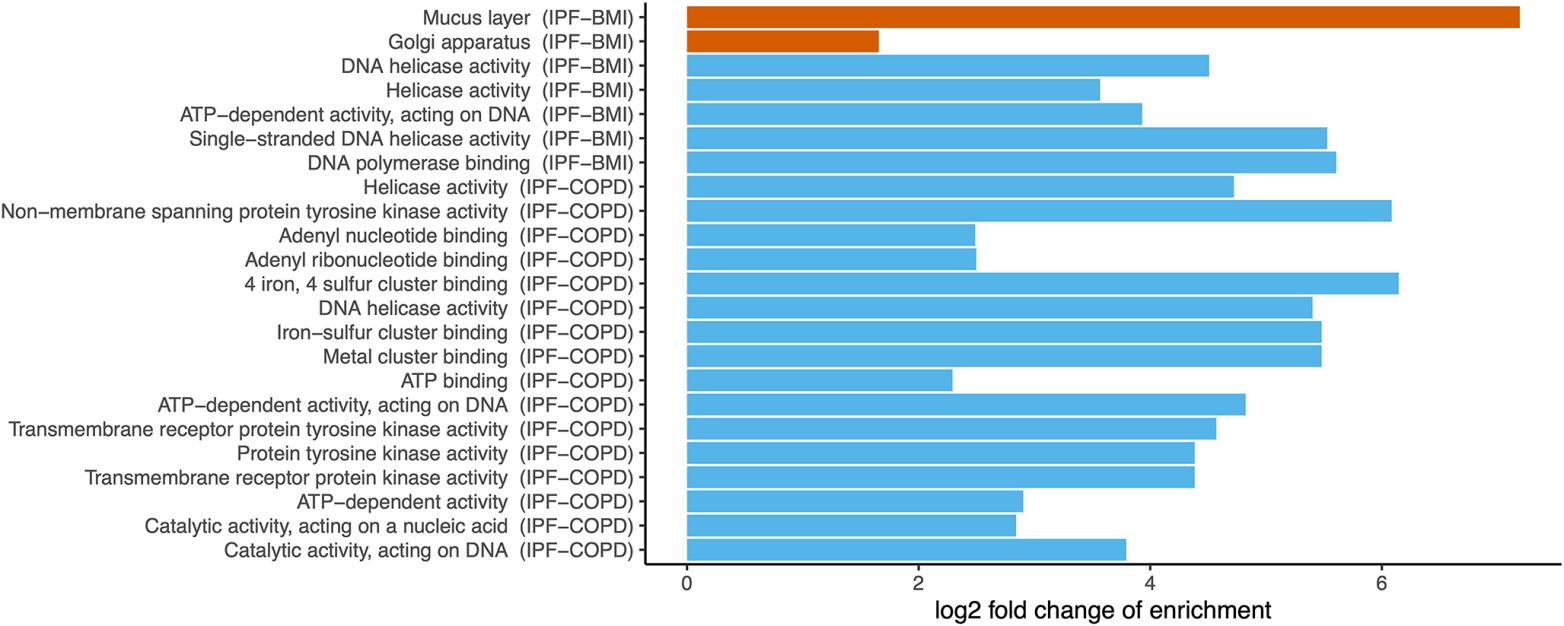
Significant pathways shared underlying IPF-BMI and IPF-COPD, respectively, using ShinyGO gene-set enrichment analysis. Significant pathways are defined by gene ontology (GO, cellular component in red and molecular function in blue), with at least two human gene annotations, and surpass the 5% FDR significance level. FDR: Benjamini–Hochberg false discovery rate. IPF: idiopathic pulmonary fibrosis. COPD: chronic obstructive pulmonary disease

In the final phase, we conducted a pathway enrichment analysis to investigate whether the sets of candidate genes identified by mBAT-combo could reveal potential biological pathways shared between IPF and each of BMI, CAD, COPD, GER, and T2D, respectively. Our analysis identified 20 FDR significant pathways (FDR < 5%; Fig. 5 and Table S6) that were notably over-represented in IPF-BMI (*n* = 7) and/or IPF-COPD (*n* = 16), with three overlapped pathways related to DNA helicase activity. Among the identified pathways, six had at least 5 gene annotations or comprised at least 20% of the pathway-specific gene annotations. This included three for IPF-BMI related to mucin synthesis and transportation and three for IPF-COPD related to ATP metabolism. All of these pathway terms exhibited robust enrichment, with at least a 3-fold increase, and one term (‘GO cellular component: Mucus layer’) reached an extraordinary fold enrichment threshold exceeding 100. Notably, this pathway was predominantly represented by the mucin gene clusters (Table S6). These findings shed light on potential biological pathways that may underlie the shared genetic architecture between IPF and its comorbidities, offering valuable insights for further investigation and therapeutic targeting.

## Discussion

This study provided previously unreported insights into the genome-wide genetic basis shared between IPF and its comorbidities. Our report utilised large-scale GWAS datasets of IPF and its comorbidities to provide robust evidence supporting the pervasive genetic factors shared between IPF and its common comorbidities, most of which demonstrated a substantially high or moderate magnitude of the genetic correlation.

Interestingly, the application of LCV model revealed a putatively partial causal effect of IPF on the development of CAD. However, this finding was not supported by three MR models (MR-Egger, weighted model and CAUSE) maybe due to their specific limitations. The MR-Egger model assumes a constant pleiotropic effect across instrumental variables, which may overestimate pleiotropy and produce highly conservative results (Burgess and Thompson 2017); the weighted mode model utilises a subset of instrumental variables with consistent effect size, which probably yields insufficient power compared to other MR models (Hartwig et al. 2017); and the CAUSE is conducted using a more extensive set of instrumental variables with p-value < 1 × 10^− 3^, which may introduce noise if the potential confounders exist (Morrison et al. 2020). Taking both LCV and multiple MR into consideration, our results suggested the existence of both pleiotropy and partial causality between IPF and CAD. This relationship might be attributed to some potential pathophysiological mechanisms underlying the two conditions. For instance, ischemia in the sub-endocardium may result from impaired oxygen delivery due to the lungs’ reduced capacity to oxygenate haemoglobin in IPF patients. Additionally, the elevation of interleukins and tumour necrosis factor-alpha in IPF patients might lead to the accumulation of oxidised low-density lipoprotein, potentially triggering proatherogenic immune responses that contribute to the development of CAD (Kizer et al. 2004; Sinha et al. 2024). Nevertheless, further studies based on larger sample sizes are required to validate this finding. Furthermore, a recent study (Zhu et al. 2023) employed MR to explore the putative causal relationships between IPF and 22 comorbidities, providing suggestive evidence for putative causalities between IPF and COPD, GER, venous thromboembolism and hypertension. While our study did not replicate these causal relationships mainly because of using a more conservative LCV model, we validated the significant shared genetic factors between IPF and its comorbidities, including GER and COPD.

The current cross-trait meta-analysis identified a list of previously unreported SNPs significantly associated with specific pairs of IPF and its comorbidities. Many of these SNPs were specific to IPF-BMI and widely distributed across 13 chromosomes, providing additional evidence for a pervasive genetic basis shared between IPF and BMI. Moreover, two SNPs (rs10947563 and rs62402061) were shared among multiple diseases (IPF, BMI and GER), indicating the common pathways among the three conditions. In addition to shared genetic evidence, several cohort studies reported that GER plays a role in driving disease progression in IPF patients through increased risk for micro-aspiration of gastric acid or other non-acid components of gastric fluids (Lee et al. 2010; Raghu and Meyer 2012). While BMI is a comorbidity of IPF, it is the low BMI that is linked to worse prognosis whereas a higher BMI is associated with better survival in IPF patients (Comes et al. 2022; Cotton et al. 2023; Kulkarni et al. 2019; Sangani et al. 2021), suggesting weight monitoring could be an effective clinical measure for individuals with severe IPF. On the other hand, low BMI is protective for GER, as reported by multiple clinical studies (Jacobson et al. 2006; Singh et al. 2013), suggesting that the negative effect of low BMI on IPF prognosis is probably not via inducing GER but via a more direct mechanism. More clinical data and epidemiological studies are required to validate our findings on a genetic level.

Based on the cross-trait GWAS summary data, we observed consistent and significant enrichment of susceptibility loci, suggesting a shared genetic basis, in the lung for IPF-CAD and the liver for IPF-COPD comorbidities. This implies a non-identical shared etiology between IPF-CAD and IPF-COPD, manifesting in different tissues. The lung enrichment for susceptibility loci in IPF-CAD is intriguing, given that CAD is a non-respiratory disease and prior research did not report significant heritability enrichment in the lung for CAD (Aragam et al. 2022). This finding suggests that the shared underlying etiology in the lung may be evident in IPF before the onset of CAD, providing further evidence for a causal effect of IPF on CAD, at least to some extent. The liver enrichment for susceptibility loci in IPF-COPD adds weight to the observed common occurrence of interactions between the lung and liver in acute respiratory diseases (Herrero et al. 2020; Mahmud et al. 2021). Additionally, when incorporating suggestive significant SNPs as input, enrichments specific to brain regions were observed for susceptibility loci in IPF-BMI. This suggests the potential involvement of brain-related pathways in this trait pair, although the predominant signals likely originate from BMI (Ndiaye et al. 2020). These findings underscore the tissue-specific nature of the shared genetic architecture between IPF and its comorbidities, providing valuable insights into the complex interplay of genetic factors across different tissues and organ systems.

Our study revealed several candidate genes. First, four mucin genes (*MUC2*,* MUC5AC*,* MUC5B and MUC6*) were shared between IPF and BMI. Mucins are large, highly glycosylated macromolecular components of secreted mucus with a role in lubricating and viscoelastic mucus (Sheng and Hasnain 2022). We further identified that the mucus layer is the most significant biological pathway shared between IPF and BMI. The expression changes of these mucin genes may result in alterations in the function, structure, and quantity of mucins, thereby altering the barrier function of mucus and affecting the mucosal inflammation in IPF and BMI (Sheng et al. 2012). Among the four mucin genes, the gene expression change of *MUC2* has been reported to be related to the increased risks for IPF and BMI through specific mechanisms. *MUC2* deficiency impairs *β*-defensin mRNA expression and peptide localisation in the gut, resulting in gut dysbiosis and endotoxin translocation (Cobo et al. 2015). Subsequently, gut dysbiosis has been linked with changes in immune responses and homeostasis in the airways, and it may have a profound consequence on interstitial lung diseases (Chioma et al. 2021). Gut dysbiosis is also a well-recognised factor contributing to the pathophysiology of obesity (Breton et al. 2022), implying that *MUC2* expression could contribute to IPF and BMI. Furthermore, *MUC2* was also observed to interact with three clinically relevant drugs (i.e., fluorouracil, chembl35482, and decitabine). Of these, decitabine, a DNA-targeted hypomethylating drug, is reported to be beneficial for managing IPF patients (Schiza et al. 2015), suggesting the potential use of epigenetic drugs in treating IPF patients with higher BMI.

In addition to the above genes, the other four genes conveying significant genetic associations in IPF and BMI are *GPC2*,* GAL3ST4*,* CNPY4* and *MBLAC4,* which contribute to secretory proteins transcription, translation, and trafficking process. *GPC2*, encoding Glypican 2, is a member of the glypican family genes that produce proteoglycan with a glycosylphosphatidylinositol anchor and is a cell surface oncoprotein in neuroblastoma (Bosse et al. 2017). Similarly, *GAL3ST4* encodes a protein member of the galactose-3-O-sulfotransferase protein family, which likely changes the typical sulfation pattern of glycans, thereby altering the physiologic functions of various glycoproteins like mucins (Qiu et al. 2021). *MBLAC1* encodes Metallo-β-lactamase Domain-Containing Protein 1, a protein not previously associated with IPF. *MBLAC1* has robust acyl-CoA thioesterase activity in vitro and could potentially play a role in regulating cellular levels of acyl CoAs, essential substrates in disulphide bond formation, glycoprotein packaging and transportation (Malgapo et al. 2021). Subsequently, *CNPY4* encodes canopy 4 and is involved in protein localisation to the plasma membrane and membrane composition (Lo et al. 2022). Collectively, these four genes are involved in regulating energy consumption, glycoprotein synthesis and secretion, suggesting that BMI-associated dysregulated carbohydrate and protein synthesis are strongly associated with IPF progression.

Interestingly, another gene, *SNRPC*, regulates the risks for IPF and four comorbidities (i.e., BMI, CAD, GER, and T2D). *SNRPC* encodes small nuclear ribonucleoprotein polypeptide C, which participates in the splicing nuclear precursor-mRNA. Supporting our observation, previous GWAS meta-analysis linked *SNRPC* with body weight regulation in the context of feeding (Ignatieva et al. 2016), obesity severity (Riveros-McKay et al. 2019) and severity of atherosclerosis (Haitjema et al. 2017), providing a genetic link of *SNRPC* to BMI, GER, T2D and CAD. Similarly, extensive proteome profiling of human lung biopsy revealed that *SNRPC* is differentially expressed in individuals with interstitial lung disease compared to healthy control (Schiller et al. 2017), demonstrating the direct link of *SNRPC* function to IPF. However, whether *SNRPC* regulates IPF via the same pathway as it does for BMI, T2D, CAD and GER is unclear. In-depth analysis and in vivo experiments that investigate the biological function of *SNRPC* gene are required to dissect the correlations.

Some drug-gene interactions also warrant attention. Four drugs, namely budesonide, fluoxetine, hesperidin and imatinib, show interactions with the genes associated with the susceptibility to both IPF-BMI and IPF-CAD (i.e., *BRSK2*, *CRHR1*, *ULK3*). They are recognised for their potent anti-inflammatory effects, particularly their anti-fibrotic properties, prompting research into their potential use in treating IPF (Chennakesavulu et al. 2018; Daniels et al. 2010; Deidda et al. 2017; Zhou et al. 2019). These four drugs are also reported to have potential efficacy in the treatment of cardiovascular conditions, such as CAD (Distler and Distler 2007; Khorasanian et al. 2023; Lofdahl et al. 2007; Roose et al. 1998), and obesity, characterised by high BMI (Breccia et al. 2013; Forno et al. 2011; Serralde-Zuniga et al. 2019; Xiong et al. 2019), possibly attributing to their anti-inflammatory properties, antioxidant activities, and/or potential metabolic effects. Moreover, another drug, lansoprazole, is known as a type of proton pump inhibitor and interacts with *MAPT,* which is associated with the risk of IPF-CAD. It is reported to reduce IPF progression (Ghebre and Raghu 2016; Ghebre 2018) and is likely relevant to alleviate heart pressure, as evidenced by a mouse model (Lin et al. 2020). These findings may offer promising prospects for drug repurposing opportunities for the treatment of IPF patients with comorbidities like overweight and CAD.

Our study has some limitations. First, all GWAS summary data used in our study were of European ancestry. Therefore, the interpretations of our findings might not apply to other populations because of the differences in the proportions of genetic variations. Second, the MHC region was excluded from our analyses because most quantitative genetics approaches cannot fully handle the complex LD structure within the MHC region. Given the MHC region’s critical role in the immune regulations, the shared genetics between IPF and its comorbidities in our study might be undervalued. Third, our study systematically evaluated the common genetic variants (with a minor allele frequency > 0.01) and did not include rare genetic variant analysis, primarily due to their low frequency and the different statistical approaches and power required. This limitation might have led to overlooking some critical shared genetic contributions to IPF and specific comorbidities, particularly in cases where rare variants play a crucial role. Among them lacks an independent European-based GWAS datasets on IPF and its comorbidities for validating the reported results. As such, our study is thought to serve as a discovery phase for investigating the shared genetic landscape between IPF and specific comorbidities. Our findings only provided suggestive mechanisms underlying IPF and its comorbidities at a statistically significant level. Further studies are requested to confirm our findings and determine the clinical relevance.

## Conclusions

In conclusion, our study provides evidence for unreported pervasive genetic correlations between IPF and its common comorbidities, a partial genetic causal effect of IPF on CAD, and tissue-specific genetic enrichments for cross-trait of IPF and specific comorbidities. Our findings also identified multiple candidate SNPs, risk genes, and their associated potential drugs and biological pathways for specific pairs of IPF and its comorbidities. Our results have enriched current knowledge of the shared genetic architecture between IPF and its common comorbidities, shedding light on improving prevention and new clinical treatment of IPF.

## Electronic supplementary material

Below is the link to the electronic supplementary material.

Supplementary Material 1

Supplementary Material 2

## Data Availability

GWAS summary data for IPF and COPD are available from http://results.globalbiobankmeta.org/. GWAS summary data for BMI are available from https://portals.broadinstitute.org/collaboration/giant/index.php/GIANT_consortium_data_files. GWAS summary data for CAD are available from http://ftp.ebi.ac.uk/pub/databases/gwas/summary_statistics/GCST005001-GCST006000/GCST005194/. GWAS summary data for GER are available from https://figshare.com/articles/dataset/GERD_GWAS_summary/8986589. GWAS summary data for lung cancer are available from https://github.com/Wittelab/pancancer_pleiotropy. GWAS summary statistics for MDD are available from https://www.med.unc.edu/pgc/download-results/. GWAS summary data for OSA are available from https://www.finngen.fi/en/access_results. GWAS summary data for stroke are available from https://www.megastroke.org/download.html. GWAS summary data for T2D are available from https://cnsgenomics.com/content/data.
